# Multimodal 3D DenseNet for *IDH* Genotype Prediction in Gliomas

**DOI:** 10.3390/genes9080382

**Published:** 2018-07-30

**Authors:** Sen Liang, Rongguo Zhang, Dayang Liang, Tianci Song, Tao Ai, Chen Xia, Liming Xia, Yan Wang

**Affiliations:** 1 Key Laboratory of Symbol Computation and Knowledge Engineering of Ministry of Education, and College of Computer Science and Technology, Jilin University, Changchun 130012, China; hawkcoder@gmail.com; 2 Advanced Institute, Infervision, Beijing 100000, China; zrongguo@infervision.com (R.Z.); songtianci1993@hotmail.com (T.S.); xchen@infervision.com (C.X.); 3 School of Mechatronics Engineering, Nanchang University, Nanchang 330031, China; 5910116336@email.ncu.edu.cn; 4 Department of Radiology, Tongji Hospital, Wuhan 430030, China; aitao007@hotmail.com; 5 Cancer Systems Biology Center, China-Japan Union Hospital, Jilin University, Changchun 130033, China

**Keywords:** multimodal deep learning, three-dimensional DenseNet model, isocitrate dehydrogenase genotype, magnetic resonance imaging, gliomas, World Health Organization grade

## Abstract

Non-invasive prediction of isocitrate dehydrogenase (*IDH*) genotype plays an important role in tumor glioma diagnosis and prognosis. Recently, research has shown that radiology images can be a potential tool for genotype prediction, and fusion of multi-modality data by deep learning methods can further provide complementary information to enhance prediction accuracy. However, it still does not have an effective deep learning architecture to predict *IDH* genotype with three-dimensional (3D) multimodal medical images. In this paper, we proposed a novel multimodal 3D DenseNet (M3D-DenseNet) model to predict *IDH* genotypes with multimodal magnetic resonance imaging (MRI) data. To evaluate its performance, we conducted experiments on the BRATS-2017 and The Cancer Genome Atlas breast invasive carcinoma (TCGA-BRCA) dataset to get image data as input and gene mutation information as the target, respectively. We achieved 84.6% accuracy (area under the curve (AUC) = 85.7%) on the validation dataset. To evaluate its generalizability, we applied transfer learning techniques to predict World Health Organization (WHO) grade status, which also achieved a high accuracy of 91.4% (AUC = 94.8%) on validation dataset. With the properties of automatic feature extraction, and effective and high generalizability, M3D-DenseNet can serve as a useful method for other multimodal radiogenomics problems and has the potential to be applied in clinical decision making.

## 1. Introduction

Gliomas are the most frequent malignant primary brain tumors in adults, and screening and diagnosing for them is still a big challenge. It can be classified into four grade levels from I to IV according to the World Health Organization (WHO) [1]. More than half of glioma patients are in low-grade status (grade II and III, known as low-grade gliomas, LGG), and a fraction of patients are in high-grade status (grade IV, known as glioblastoma multiform, GBM). Glioblastoma multiforms have also been classified as primary or secondary glioblastomas clinically. In 2008, a multi-group collaboration found that *IDH1* mutations occurred in 12% of glioblastomas [2], and subsequent researchers observed *IDH1* mutations in 50–80% of LGGs and secondary GBM [3]. Before these observations, the isocitrate dehydrogenase (*IDH*) genotype had never been linked to cancer [4]. Isocitrate dehydrogenase is a general term for *IDH1* and *IDH2* gene, which encode cytosolic *IDH1* and mitochondrial *IDH2*, respectively. These are major enzymes in citric acid cycle processing that play a key role in the defense against oxidative stress [5]. Normally, these gene products convert isocitrate into α-ketoglutarate [6]. However, when *IDH* is mutated, the isocitrate will be converted to 2-hydroxyglutarate, which can inhibit the activity of man α-ketoglutarate-dependent dioxygenases, including, but are not limited, to histone demethylases [7]. Several lines of research however, have shown that *IDH1* mutations were early events in the development of gliomas [8] and had a different impact on gliomas. Previous studies demonstrated that *IDH1* mutations frequently occurred in grade II and III gliomas and secondary GBM glioblastoma, but rarely in primary GBM [9]; whereas *IDH2* mutations occur in fewer than 3% of glial tumors. Hartmann et al. provided evidence that patients with *IDH1* wild-type anaplastic astrocytomas exhibit a worse prognosis than *IDH1* mutated [10]. Nobusawa et al. pointed out that *IDH1* mutation could be a molecular signature and predictive factor of secondary glioblastomas [11], which may serve as biomarkers for assessing tumor progression and treatment. Among all molecular alteration types in gliomas, *IDH* genotype is the most important, as patients with the *IDH* gene mutation have significantly longer survival time than patients of *IDH* wild-type, which is independent of WHO grade [12,13].

The *IDH* genotype is often identified by immunohistochemistry and DNA sequencing techniques [14,15]. These methods rely on biopsies and invasive surgeries and assess genotype information derived from small portions of tumor tissues, which may provide a fractional and sometimes biased reading of the whole tumor. In clinical practice, pre-treatment identification of *IDH* genotype can help guide a clinical decision. However, it is still a challenging problem to predict *IDH* genotype non-invasively. Previous studies have reported an association between radiology imaging features and *IDH* genotype within gliomas. Qi et al. conducted a retrospective study on 193 patients and found that *IDH*-mutated gliomas were more frequently confined to a single lobe and more likely exhibit a unilateral pattern of growth, sharp tumor margins, homogenous signal intensity, and less contrast enhancement on magnetic resonance imaging (MRI) images [16]. In a study on 202 patients [17], imaging features like larger tumor size, frontal lobe localization, and presence of cysts and satellite lesions, were reported to be better recognized *IDH* mutations from *IDH* wild-type. Zhou et al. conduct a radiomics model using texture features and visually accessible Rembrandt images (VASARI) annotations features to predict *IDH1* mutations and found that the texture feature achieved a higher accuracy than with the VASARI features [18].

Recently, radiogenomics is becoming a fast-developing research area [19,20], combining radiology imaging with genomics data to help improve clinical diagnosis. Genomics data, such as gene expression profiling and genotyping, play an important role in precision medicine, especially in cancer screening and therapy [21,22,23]. Radiology imaging techniques like computed tomography (CT), X-ray, and MRI, on the other hand, can provide a more thorough view of the entire tumor and be used to monitor the progression of a tumor. Furthermore, compared to genomics methods such as mass spectrometry-based mutation genotyping, radiology imaging is noninvasive and cost-effective, and is readily available in clinical procedures. Therefore, it will be valuable to utilize radiology imaging to predict genotype status. Previous literature has shown that radiology imaging can be a potential tool for the prediction of genotypes, and the fusion of multi-modality data can provide complementary information to further enhance prediction accuracy [24,25,26,27].

In clinical practice, when diagnosing a disease, doctors often consult data from multiple modalities such as X-ray images, CT/MRI scans, clinical data and so on. For instance, radiologists often read both CT and MRI scans to evaluate acute stroke [28,29]. Multiple sequences of MRI images (native (T1), and post-contrast T1-weighted (T1Gd), T2-weighted (T2), and T2 fluid attenuated inversion recovery (FLAIR)) are often produced to assess and diagnose glioma tumors [26,30,31]. Recently, many researchers have proposed methods to predict genotypes from multimodal image data. Zhang et al. [26] manually extracted features from multimodal MRI images with traditional radiomics methods, and then employed a random forest algorithm to predict *IDH* genotype based on handcrafted image features and clinical data. Although this method has better interpretability, as authors used radiomics features to perform genotype classification, its handcrafted features are not comprehensive. Chang et al. [25] first trained a residual convolutional neural network for each MRI image modality (T1, T1Gd, T2, FLAIR) and then built a logistics regression model to integrate outputs from each of the four neural networks and make predictions. Chang et al.’s technique promoted feature extraction with deep learning; nevertheless, their two-stage modeling procedure can be improved by fusing multimodal information at the training stage and modifying the model to better fit three-dimensional (3D) volume data.

In the same time, with rapid advancement in computer vision, the convolutional neural network (CNN) is becoming an important tool in medical imaging analysis [32]. In 1998, LeCun et al. proposed the LeNet-5 neural network [33] to recognize hand-written numbers. A dozen years later, Krizhevsky et al. conducted AlexNet [34] to win the ImageNet Large-Scale Visual Recognition Challenge (ILSVRC) in 2012, when graphics processing unit (GPU) was first implemented to parallelize neural networks with cross-connections. GoogLeNet [35] was inspired by LeNet but proposed a novel inception module, which was based on several small convolution networks to reduce the number of parameters. Simonyan et al.’s VGGNet [36] has very uniform architecture, which is often used for extracting features from images, but its large numbers of parameters can be challenging to manage. In 2015, a novel architecture network with a characteristic of residual connections called residual neural network (ResNet) was introduced by He et al. [37]. ResNet was able to train a 152 layers network with lower complexity. Followed this connectivity pattern benefit and further reducing the number of model parameters, Huang et al. proposed the densely connected convolutional networks (DenseNet) [38] with multiple dense block elements that connect each layer to every other layer in a feed-forward fashion. DenseNet requires less computation and less model complexity, and achieves significant improvements over the state-of-the-art CNN on most benchmark tasks. However, we need to make some improvements for it if we apply DenseNet in 3D multimodal medical images, like multiple sequence MRI data, as it is suited for two-dimensional (2D) images and single modality input.

In this paper, we developed a multimodal 3D deep learning model based on 3D DenseNet framework, called multimodal 3D DenseNet (M3D-DenseNet), to predict *IDH* genotypes with multiple sequences of MRI images. Specifically, we used DenseNet as the building block to reduce overfitting and model complexity. In addition, a multi-channel technique was employed to integrate multimodal information by sharing parameters. The rest of the paper was organized as follows: Section 2 introduced our experiments data description and its preprocessing and described M3D-DenseNet architecture and its implements in detail. In Section 3, we first conducted an experiment to predict *IDH* genotype with multimodal MRI data and then applied transfer learning to tumor grade prediction to further evaluate the generalizability of our model. In Section 4, we discussed our experimental results and the implication of the current methodology for future radiogenomics research. In the last section, we gave a conclusion for this manuscript.

## 2. Materials and Methods

### 2.1. Data Description

#### 2.1.1. BRATS-2017 Dataset

The dataset of multimodal brain tumor segmentation challenge 2017 (BRATS-2017) [30,39] comprises of clinically-acquired 3 Tesla (T) multimodal MRI scans of GBM and LGG, and all ground truth labels were manually-reviewed by expert board-certified neurobiologists. Glioblastoma multiform and LGG refer to the same brain tumors called gliomas, with high- and low-grades respectively. Each patient’s data included: (i) *IDH* genotype, acquired from The Cancer Genome Atlas breast invasive carcinoma (TCGA-BRCA) dataset; (ii) tumor grade status, where we labeled all LGG tumors as low grade and GBM as high grade; (iii) four MRI scans including T1, T1Gd, T2, and T2-FLAIR volumes; (iv) tumor ground truth labels (called mask data); (v) clinical data such as age and sex, which can be fused into model to improve model prediction ability. A total of 167 glioma patients (102 GBM patients and 65 LGG patients) were collected and five-fold cross-validation is conducted to evaluate model performance. The clinical characteristics of all patients are shown in Table 1.

#### 2.1.2. The Cancer Genome Atlas Breast Invasive Carcinoma Dataset

The cancer genome atlas (TCGA) [40] is a project funded by the US government to catalog the genetic mutations responsible for cancer, using genome sequencing and bioinformatics techniques. This dataset contains 33 cancers with various data types, including gene expression profiling, copy number variation profiling, single nucleotide polymorphism (SNP) genotyping [41], and so on. Since the GBM and LGG tumor datasets of TCGA came from the same group of patients in BRATS-2017, we could acquire patients’ multimodal MRI image data from BRATS-2017 and obtain patients’ corresponding *IDH* mutation status data from the TCGA dataset. To determine whether one gene is mutated or not, the TCGA dataset uses four methods in parallel: MuSE [42], MuTect2 [43], SomaticSniper [44], and VarScan2 [45]. In this paper, we considered a gene to be in mutation status when two or more of these four methods indicate that a gene is mutated.

### 2.2. Data Preprocessing

**Figure 1:**
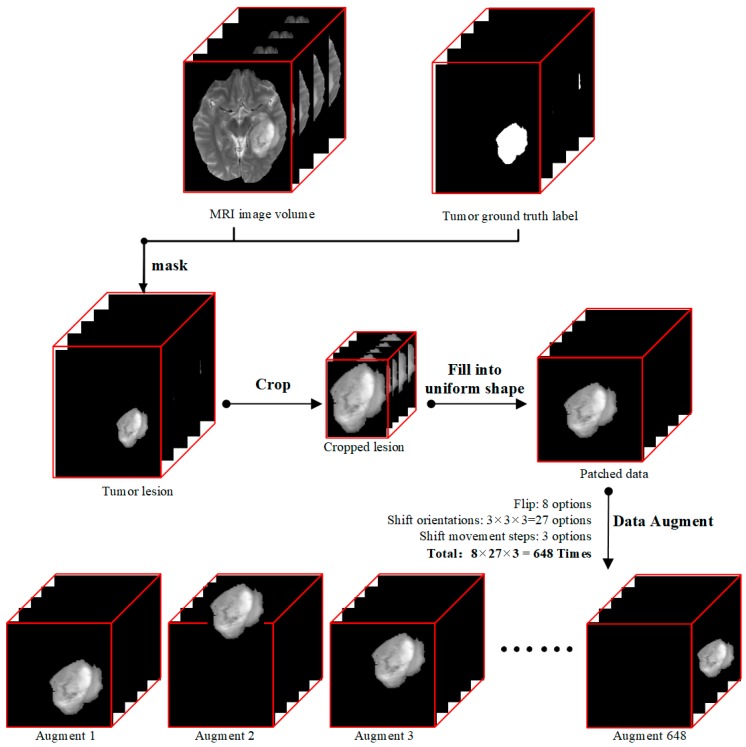
Data preprocessing flow diagram. (i) We first masked data with a magnetic resonance imaging (MRI) image volume data and ground truth label, (ii) and then cropped the lesion to a small cube, (iii) followed by filling into a uniform shape to produce our input data called patched data. (iv) At last, considering small data quantity and model overfitting, we did data augmentation for each patched data 648 times by flipping and shifting.

Our data preprocessing procedure followed the diagram showed in Figure 1. Each modality of T1, T2Gd, T2, and FLAIR MRI volume data, which had the shape of 155 × 240 × 240 pixels, was processed through the following four steps. (i) We derived tumor lesion volume data (155 × 240 × 240 pixels) by doing a matrix multiplication between MRI image volume and mask data so that only pixels of tumor lesions were kept, and all other pixels were set to zero. (ii) Then we cropped out the 3D tumor lesions to their actual sizes. (iii) As the sizes of tumors were different and our deep learning model required uniform input size, we patched zeroes outside of cropped tumor lesions to produce data patches of the same size of 112 × 112 × 142 pixels. The data patch shape was determined by the largest dimension values of all lesions in the dataset because radiomics literature has shown that tumor size is a key factor for the prediction of tumor genotype [46]. (iv) Since the dataset was small, we used a data augmentation technique to expand our training data.

### 2.3. Multimodal Three-Dimensional DenseNet

**Figure 2:**
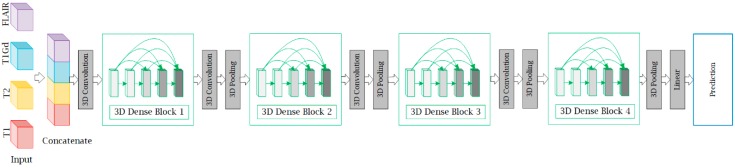
A schematic illustration of multimodal three-dimensional DenseNet (M3D-DenseNet). Our M3D-DenseNet takes four MRI sequences (l, w, h) as input and then concatenates the four modalities as a four-dimensional matrix (4, l, w, h). All convolutions and poolings in the network are 3D operations, and are the same in the four dense blocks. T1: native; T1Gd: post-contrast T1-weighted ; T2: T2-weighted; FLAIR: T2 fluid attenuated inversion recovery.

In this section, we describe the structure of M3D-DenseNet, which is proposed for genotype prediction based on multiple MRI sequences. Our M3D-DenseNet took four single-modality MRI sequences (T1, T2, T1Gd, FLAIR) as input and then concatenated these modalities as a four-dimensional matrix (4, l, w, h), where “4” represented four channels and (l, w, h) represented the shape size (length, weight, height) of each single-modality MRI sequence data. To fit the 3D natural instinct of MRI images, we applied a 3D deep learning framework to our model, which was modified from DenseNet. In Figure 2, we illustrated our neural network architecture through a schematic diagram. In addition, Table 2 shows the M3D-DenseNet network architecture information with different depth of 121, 161, 169, and 201 layers.

#### 2.3.1. Three-Dimensional Deep Learning Framework

Radiology imaging often produces 3D volume data. For example, CT and MRI images usually contain multiple sequences, and each sequence has three representative orthogonal views of axial, coronal, and sagittal planes. The CNN deep learning frameworks, however, is usually constructed to solve computer vision problems within 2D images, like classification [34], detection [47], and segmentation [48]. With this intention, we modified the deep learning framework so that it directly takes volume data as input and can better fit 3D radiology imaging. Although a 3D deep learning framework makes it more straightforward to train volume data, it brings an intrinsic problem of a much larger set of parameters, and thus a higher propensity of overfitting when the dataset is small. To overcome this potential problem, we selected DenseNet, which is known for its advantage of reducing model complexity, as our backbone network.

#### 2.3.2. DenseNet

DenseNet is a convolutional neural network that connects each layer to every other layer in a feed-forward fashion. For each layer, the feature-maps of all preceding layers are used as inputs, and its own feature-maps are used as inputs into all subsequent layers [38]. DenseNet introduces a 1×1 convolution as a bottleneck layer before each convolution layer to eliminate the number of feature maps. Each dense block contains the bottleneck structure and dimension reduction in transition layers, which makes parameters more efficiently, and furthermore reduces model complexity. The computational efficiency of DenseNet has been proven by works such as He et al. [37] and Szegedy et al. [49]. In our M3D-DenseNet model, we modified the original 2D DenseNet to a 3D DenseNet by converting all 2D convolution and pooling operators to their 3D versions. Since our dataset had 167 samples which are not large enough, using DenseNet as building blocks could reduce the risk of overfitting and speed up training.

#### 2.3.3. Multi-Channel Technique

In this paper, we focused on homogeneous multimodal data, which were obtained from the same imaging method but with different parameters. Take MRI scans as an example, sequences including T1, T2, T1Gd, and FLAIR can be produced from a patient’s single brain scan. Unlike heterogeneous multimodal data that are usually different in data structure such as image, text and audio, each modality of homogeneous multimodal data has almost the same data structure. With this property of homogeneous multimodal data, we used a multi-channel technique that stacks different modalities as different channels. This method can facilitate different modalities to share parameters during the training stage and fuse multi-modality information at all model depths.

### 2.4. Implementation Details

Our model implementation was performed under the MXNet (version 1.0, Apache Software Foundation, Forest Hill, MD, USA) deep learning framework [50]. During training, the *IDH* mutation probability of each sample was calculated by a softmax alogorithm classifier, and binary cross-entropy was chosen as the objective function of our network. We used the rectified linear unit (ReLU) activation function in each layer and applied a batch normalization technique before each ReLU layer. The weights of our network were optimized by the stochastic gradient descent (SGD) method with a mini-batch size of eight. The learning rate was set to 0.01 with a momentum coefficient of 0.9, and a decay rate of 0.1 every 50 epochs. We trained our model on four NVIDIA Titan X GPUs (NVIDIA, Beijing, China).

#### 2.4.1. Data Augmentation

In order to avoid overfitting, for each data patch with the shape of 112 × 112 × 142 pixels, we used the following two data augmentation methods: (i) We flipped the data patch along three orthogonal dimensions (coronal, sagittal, and axial position), a combination of eight transformations. (ii) For each data patch, we shifted the tumor along the three orthogonal dimensions. On each dimension, the shift operation took one of the three options (move in the positive direction, move in the negative direction, and stay at the same place), and each shift movement took one of three lengths (1/3, 2/3, or 3/3 % of the distance between the margin of tumor lesion and margin of the data patch). In total, each data patch could be augmented to a maximum of 8 × 3 × 3 × 3 time = 648 times.

#### 2.4.2. Parameter Initialization

As the pixel distribution of tumor and non-tumor tissues can vary across regions even in the same brain, we trained our model from scratch with the Xavier initialization method [51]. Xavier initializes the model weights from a distribution with zero mean and Var(W) variance:

(1) $$Var\left(W\right)=\frac{2}{n_{in}+n_{out}},\;$$

where $n_{in}$ and $n_{out}$ represent the number of input and output hidden nodes of each layer. Xavier initialization assumes that activations are linear; however, our model used the nonlinear ReLU as the activation function. Considering this weakness, we added a batch normalization [52] layer before each ReLU layer to normalize each training batch.

#### 2.4.3. Transfer Learning

In practice, researchers often apply transfer learning technique to fine-tune their new model with pertain model [53,54]. However, no pre-trained model was available for our dataset (3D medical MRI data) and model (multimodal deep learning framework). Although transfer learning was not applicable in our first prediction task, we were able to transfer parameters pre-trained in IDH prediction experiment (Section 3.1) to our second task (Section 3.3) and fine-tune the model.

#### 2.4.4. Training Tricks

During model training, we used two tricks to prevent overfitting and to improve training speed. (i) We applied online stochastic data augmentation. This was because we knew that each modality of the data patch could have 648 kinds of augmentation, which would result in a huge input and output (IO load during training if we generated all data augmentations offline. In the following actual experiments, we conducted data augmentation online, and stochastically selected one augmentation type out of the 648 forms for each data patch during each mini-batch training. This trick helped us to expedite our model training speed in each epoch, and effectively avoided overfitting. (ii) We shuffled the training dataset sample list in each training epoch because the model may learn to remember the order of prediction labels when the dataset is small.

### 2.5. Evaluation Criteria and Measurement

In this section, we introduced four metrics to evaluate the prediction performance of our model, which were: overall accuracy (*ACC*), specificity (*SP*), sensitivity (*SN*), and area under the curve (AUC), respectively. In our prediction experiments, we defined *TP* (true positive) as the quantity of the given label that is positive, and the predicted result is also a positive; we defined *TN* (true negative) as the quantity of the given label that is negative, and the predicted result is also a negative; we defined false positive (*FP*) as the quantity of the given label that is negative, but the predicted result is positive; and defined false negative (*FN*) as the quantity of the given label that is positive, but the predicted result is negative. Specificity was the true negative rate that is correctly identified, as in Equation (3). Sensitivity was the positive predictions that were actually positive, as in Equation (4). Overall accuracy was the fraction of total samples that were correctly identified, as in Equation (2). Area under the curve calculated the area under the receiver operating characteristic (ROC) curve, which equaled the probability that a randomly chosen positive example ranked above a randomly chosen negative example.

(2) $$ACC=\frac{TN+TP}{TN+TP+FP+FN}\;$$

(3) $$SP=\frac{TN}{TN+FP}\;$$

(4) $$SN=\frac{TP}{TP+FN}\;$$

Above all, we described our experiment materials and its preprocessing steps, and described our proposal for the M3D-DenseNet network architecture. In this article, we conducted three experiments to predict *IDH* genotype, compare the performance of single-modality and M3D-DenseNet, and evaluate our model’s generalizability by predicting WHO grade status, respectively. In addition, some implementation details and evaluation measurements were also represented. In the next section, we will show the results of these experiments.

## 3. Results

### 3.1. Isocitrate Dehydrogenase Genotype Prediction Experiment

**Figure 3:**
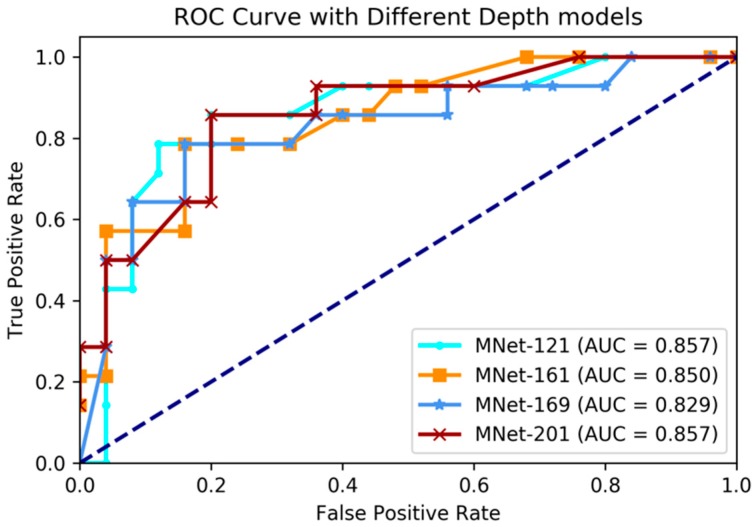
Comparing receiver operating characteristic (ROC) curve of different depth layer models on *IDH* genotype prediction experiments. AUC: area under the curve.

In this experiment, we applied our proposed M3D-DenseNet model to predict *IDH* genotype on the BRATS-2017 dataset with 167 glioma patients, whose genotype information was obtained from the TCGA-BRCA dataset. To compare the effectiveness using various network depths, four models (121, 161, 169, and 201 layers M3D-DenseNet, as Table 1) were performed in this experiment. We also conducted five-fold cross-validations to evaluate each of our models. The final result was the average of the five cross-validations. Table 3 shows the *IDH* genotype prediction performance between different layer models. Figure 3 shows the comparative ROC curve of the four models.

The experimental results showed that our proposed method had great prediction performance on *IDH* genotype prediction, and also showed robust performances on different layer depths. The best performance was achieved with accuracy 84.6%, sensitivity 78.5%, specificity 88.0%, and AUC 85.7% on the training dataset when M3D-DenseNet depth was set to 161 layers, and with an accuracy of 84.6%, sensitivity 78.5%, specificity 88.0%, and AUC 85.7% on the validation dataset when M3D-DenseNet depth was set to 121 layers.

### 3.2. Comparing Single-Modality and Multi-Modality Model Experiment

**Figure 4:**
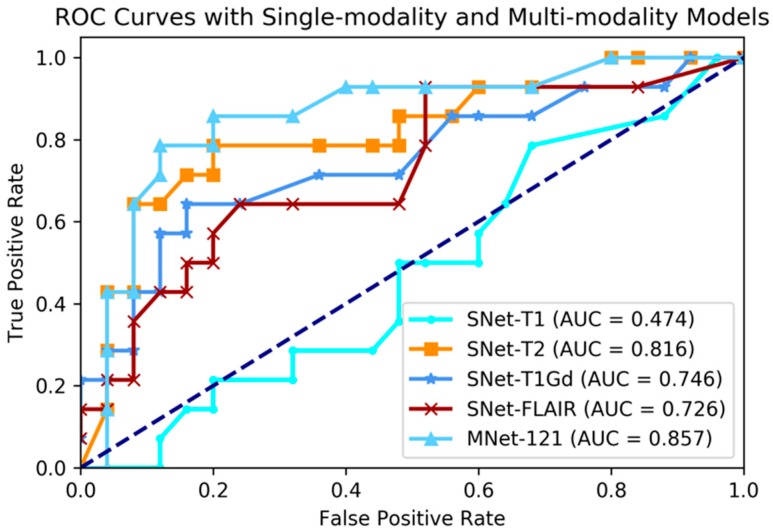
Comparing ROC curve between single-modality and multi-modality models.

**Figure 5:**
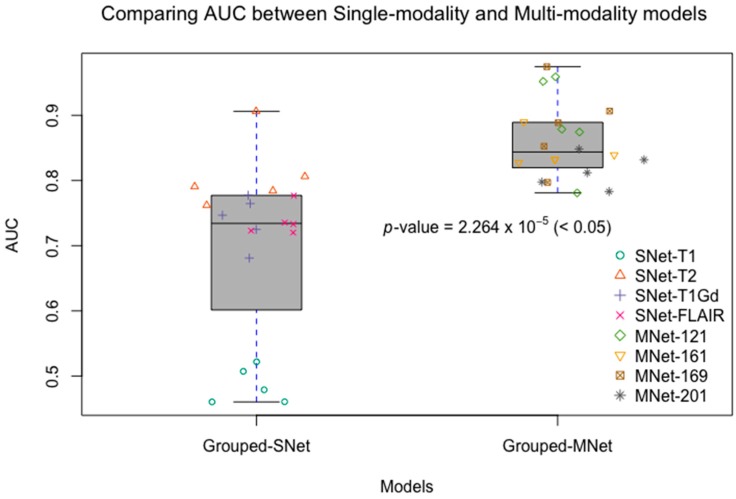
Comparing AUCs between single-modality and multi-modality models. Grouped-SNet: grouped single-modality models, Grouped-MNet: grouped multi-modality models. We grouped the results of five-fold cross-validation from all single-modality models as Grouped-SNet, and also grouped the results of five-fold cross-validation from all multi-modality models as Grouped-MNet. The student’s *t*-test result comparing Grouped-SNet and Grouped-MNet is statistically significant.

Although recent research has shown that the fusion of multi-modality data can provide complementary information to enhance prediction accuracy, there have not been systematic studies that examine the performance differences between single-modality and multi-modality models. In this experiment, we conducted four single-modality models, which are 3D DenseNet. Each single-modality model was similar to M3D-DenseNet, but the input portion, which was replaced by only one modality data from T1, T2, T1Gd, and FLAIR. In this comparative experiment, the depth of all the model was set to 121 layers. Table 4 shows the performance between single-modality and multi-modality model. Figure 4 shows the comparative ROC curve of the above five models. For the statistical comparison of single-modality model and multi-modality model, we grouped results of five-fold cross-validation from all single-modality models as Grouped-SNet, and grouped results of five-fold cross-validation from all multi-modality models as Grouped-MNet. Then, we conducted a student *t*-test to compare the difference between Grouped-SNet and Grouped-MNet with the null hypothesis that the two groups’ distributions were the same. Figure 5 shows the statistical test result.

The experimental result shown that multi-modality model was better than single-modality model, as the best accuracy of single-modality model on the validation dataset was 74.4% (AUC = 81.6%), nevertheless, the accuracy of multi-modality model on the validation dataset was 84.6% (AUC = 85.7%). By conducting a student *t*-test between the two kinds of models with the result of *p*-value = 2.264 × 10^−5^ (<0.05), we could conclude that multi-modality models were significantly better than single-modality models. On the other hand, among in the four single modalities of T1, T2, T1Gd, and FLAIR, the prediction ability of each modality model was discriminatory. With this result, we could know that the T2 modality MRI had the best *IDH* genotype prediction ability and conversely, T1 was the worst.

### 3.3. Evaluating the Generalizability of M3D-DenseNet

**Figure 6:**
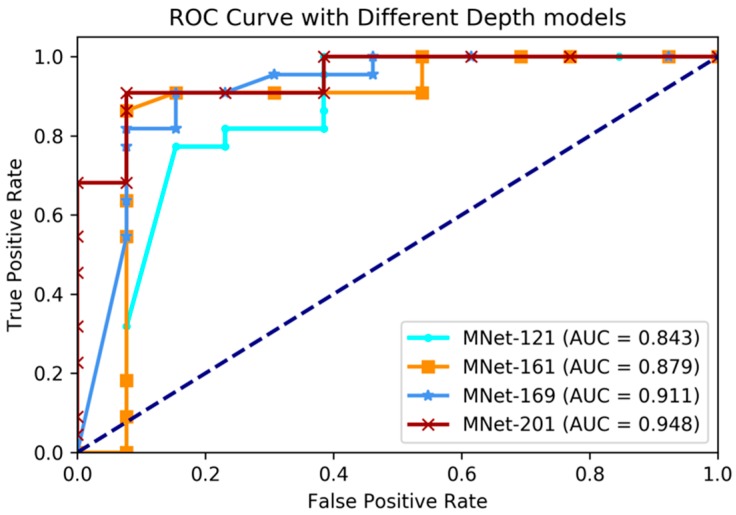
Comparing ROC curve of different depth layer models on World Health Organization (WHO) grade prediction experiments.

For the purpose of evaluating our model’s generalizability, we applied our model to predict WHO grade status, which was low-grade or high-grade. Grade status is a measurement of how malignant a tumor is. Its grading systems differ depending on the type of cancer, but it often has four degrees from benign to malignant: grade I up to grade IV. In this experiment, we regarded grade II and grade III as low-grade and regarded grade IV as high-grade. We conducted this experiment on the same BRATS-2017 and TCGA-BRCA datasets with the same experiment settings. During the model training stage, we applied transfer learning techniques to decrease training time and enhance learning accuracy. We firstly initialized our model with parameters obtained in the *IDH* genotype prediction experiment, and then fine-tuned the model to predict low-grade versus high-grade. Table 5 shows the WHO grade status prediction performance between different layer models. Figure 6 shows the comparing ROC curves of the above models.

The experimental results showed that our proposed method had great prediction performance on WHO grade prediction, and also showed robust performances on different layer depth models. The best performance was achieved when the depth was set to 201 layers; with this condition in the validation dataset, the accuracy was 91.4%, sensitivity 92.3%, specificity 92.3%, and AUC 94.8%. Comparing with the performance of four different depth models, we found that the deeper models performed with greater accuracy. Above all, it was proven that our M3D-DenseNet model had high generalizability on the prediction of WHO grade status.

## 4. Discussion

This study presents a multimodal 3D deep learning network architecture for prediction *IDH* genotype in glioma patients. In future work, we will try to involve more modality data, including both MRI and CT. Notably, we did not consider clinical information, like age and gender, since the goal of this work was to explore the relationship between multimodal MRI imaging data and *IDH* genotype through a deep learning architecture. As for integrating clinical information, Zhang et al. [26] used a random forest algorithm based on two kinds of data: clinical variables and image features extracted from multimodal MRI images by a traditional radiomics method. They used a dataset of 120 patients and achieved 89% accuracy (AUC = 0.92) on the validation data. Chang et al. [25] first trained a residual convolutional neural network on each MRI modality data and then built a logistics regression which combined outputs of each single modality network to predict *IDH* genotype. They used a dataset including 496 patients from three different sources. One point needed to be highlighted is that the performances of these two methodologies without involving clinical information were inferior to our models. Our future works also will focus on studying what kind of clinical information should be included to further boost the performance of our model.

Deep learning researchers tend to believe that deeper networks have a higher prediction power [35,38]. In experiments of Section 3.1 and Section 3.3, we made predictions with different network depths to prove them on our M3D-DenseNet model. Table 1 shows the detail network architectures with different network depths. In the *IDH* genotype prediction experiment, the accuracies on the validation data were 84.6% (AUC = 85.7%), 82.1% (AUC = 85.0%), 82.1% (AUC = 82.8%), and 76.9% (AUC = 85.7%) when the network depth was 121, 161, 169, and 201 layers, respectively. The *ACC* scores of four depth models showed differently, where the largest accuracy score was 84.6% when the network depth was 121 layers. Nevertheless, the AUC scores were not so different, so that we can know that the depth of our model did not affect the *IDH* genotype prediction result severely. However, in the WHO grade status prediction experiment, the deepest (201 layers) M3D-DenseNet produced the highest accuracy, followed by the 169-layers model, which indicates that our models with deeper networks perform significantly better. Comparing the performance of the above two experiments, it shown that the deeper networks did not always perform better. To compare the performance between multi-modality and single-modality model, we also conducted four experiments for each MRI modality data. Except for the model input portion, each single modality model was the same with M3D-DenseNet architecture. It catches one MRI volume data (1 × 112 × 112 × 142 pixels) as input, whereas the multimodal model concatenates four MRI volume data (4 × 112 × 112 × 142 pixels) as input. With the result on both prediction experiments, we found that single modality models have different performances however, and significantly inferior performance to multimodal models.

In Section 3.3, we applied the classification of WHO grade status to evaluate the ability of our model’s generalizability, as it has valuable insight in cancer diagnosis and prognosis. High-grade gliomas are highly vascular tumors and are easier to infiltrate. They have a worse median overall survival time of 15 months [55]. For low-grade gliomas, they grow slowly and can be followed without treatment until they cause symptoms. Their ten-year relative survival rate is 47% [56] and their median survival time is 11.6 years [55]. Some research has been reported on the correlation between grade and medical image features within gliomas [56]. Wiestler et al. performed a machine learning model on 37 glioma patients to predict WHO grade with multiple MRI modalities including relative oxygen extraction fraction (rOEF) and perfusion imaging [57]. They achieved a high AUC with 0.944 and found the three most important imaging features within WHO grades prediction: standard deviation of T1-weighted contrast enhanced signal, maximum regional oxygen extraction fraction (rOEF) value, and cerebral blood volume standard deviation. Garzín et al. predicted tumor grade by acquired 74 gliomas patients with multiple MRI images [58]. Their experiments showed that the presence/absence of enhancement paired with kurtosis of the FM (first moment of the first-pass curve) have high predictive value for WHO grade.

Methodologically speaking, our M3D-DenseNet has three explicit advantages. Firstly, compared to Zhang et al.’s radiomics method of extracting handcrafted features, our model can automatically extract a much larger number of features, which is more efficient [26]. Secondly, in contrast with Chang et al.’s two-stage training, our method adopts an end-to-end training scheme, and this simple procedure produced better results than Chang et al. (their results when clinical data were not incorporated) [25]. Thirdly, we applied transfer learning and fine-tuned the *IDH* genotype prediction model to predict tumor grade status, whose high prediction accuracy shows the generalizability of our methodology. Despite its satisfactory outcomes in the two prediction experiments, our model also has three limitations that can be improved in future work. Firstly, we only have data from 167 patients and a total of 668 MRI sequences. Although we used data augmentation methods to expand the training set, the actual samples model learned were still quite small. Secondly, our multimodality model fused all four sequences of MRI scans without considering the correlations among sequences. A more careful comparison of multimodality data versus single modality would help us find the most optimal combination of multiple modalities. Such study would provide us a more qualified understanding of the benefit of multimodality data. We can borrow some of the latest ideas such as non-local neural networks [59] and deep canonical correlation analysis (DCCA) networks [60], as our primary focus was on the demonstration of M3D-DenseNet rather than methods to improve neural network’s prediction power. Thirdly, we only considered homogenous multimodality data in the current paper. In the future, researchers can fuse the modalities of heterogeneous data sources such as CT images, blood exam results, and other clinical data to build a more comprehensive multimodal prediction model that can better support clinical decisions.

## 5. Conclusions

In this study, we proposed a novel network architecture to predict *IDH* genotype based on multimodal MRI imaging data, which called M3D-DenseNet. It applied a 3D deep learning framework using DenseNet as the building block, and utilized a multi-channel technique to integrate multimodal data information. To evaluate its generalizability, we fine-tuned the same model to predict WHO grade status. Both experiments achieved high prediction accuracy. Our M3D-DenseNet can train by end-to-end, automatically extract features from multi-modality images, and have greater generalizability. With further validation on more data, our models for *IDH* genotype prediction and WHO grade status prediction may have the potential to be a useful methodology that can be extended to other multi-modal radiogenomics problems and serve as a decision-making tool to help doctors make better treatment plans.

## Figures and Tables

**Table 1:** Clinical characteristics of the patients.

Clinical Features	Value
No. of patients	167
Age, mean ± SD	52.4 ± 15.5
<30	18 (10.8%)
30–60	90 (53.9%)
60–80	55 (32.9%)
≥80	3 (1.8%)
Sex	
Male	90 (54.2%)
Female	74 (45.8%)
Tumor Grade	
Low-grade (grade II, III)	65 (38.9%)
High-grade (grade IV)	102 (61.1%)
*IDH* genotype	
Mutant	53 (31.7%)
Wild-type	114 (68.3%)

*IDH*: isocitrate dehydrogenase; SD: standard deviation.

**Table 2:** Multimodal three-dimensional DenseNet architecture.

Layers	Output Size	M3D-DenseNet-121	M3D-DenseNet-161	M3D-DenseNet-169	M3D-DenseNet-201
Convolution	56×56×72	7×7×7 conv, stride 2			
Pooling	28×28×36	3×3×3 max pool, stride 2			
3D Dense Block (1) *	28×28×36	$\left[\begin{array}{c} 1\times 1\times 1\;\mathrm{conv}\\ 3\times 3\times 3\;\mathrm{conv} \end{array}\right]\times 6$			
Transition Layer (1) *	14×14×18	1×1×1 conv			
	14×14×18	2×2×2 average pool, stride 2			
3D Dense Block (2) *	14×14×18	$\left[\begin{array}{c} 1\times 1\;\mathrm{conv}\\ 3\times 3\;\mathrm{conv} \end{array}\right]\times 12$			
Transition Layer (2) *	14×14×18	1×1×1 conv			
	7×7×9	2×2×2 average pool, stride 2			
3D Dense Block (3) *	7×7×9	$\left[\begin{array}{c} 1\times 1\times 1\;\mathrm{conv}\\ 3\times 3\times 3\;\mathrm{conv} \end{array}\right]\times 24$	$\left[\begin{array}{c} 1\times 1\times 1\;\mathrm{conv}\\ 3\times 3\times 3\;\mathrm{conv} \end{array}\right]\times 36$	$\left[\begin{array}{c} 1\times 1\times 1\;\mathrm{conv}\\ 3\times 3\times 3\;\mathrm{conv} \end{array}\right]\times 32$	$\left[\begin{array}{c} 1\times 1\times 1\;\mathrm{conv}\\ 3\times 3\times 3\;\mathrm{conv} \end{array}\right]\times 48$
Transition Layer (3) *	7×7×9	1×1×1 conv			
	3×3×4	2×2×2 average pool, stride 2			
3D Dense Block (4) *	3×3×4	$\left[\begin{array}{c} 1\times 1\times 1\;\mathrm{conv}\\ 3\times 3\times 3\;\mathrm{conv} \end{array}\right]\times 16$	$\left[\begin{array}{c} 1\times 1\times 1\;\mathrm{conv}\\ 3\times 3\times 3\;\mathrm{conv} \end{array}\right]\times 24$	$\left[\begin{array}{c} 1\times 1\times 1\;\mathrm{conv}\\ 3\times 3\times 3\;\mathrm{conv} \end{array}\right]\times 32$	$\left[\begin{array}{c} 1\times 1\times 1\;\mathrm{conv}\\ 3\times 3\times 3\;\mathrm{conv} \end{array}\right]\times 32$
Classification Layer	1×1×1	3×3×4 global average pool			
		2D fully-connected, softmax			

Note: The growth rate parameter of 3D DenseNet is set to 32. Each conv layer shows in the table represents the layer sequence BN-ReLU-Conv (BN: batch normalization layer, ReLU: rectified linear unit activation layer, Conv: convolutional layer). * Numbers refer to Figure 2, 3D Dense Block 1, 2, 3 and 4, respectively.

**Table 3:** Isocitrate dehydrogenase genotype prediction performance.

	Training Dataset				Validation Dataset			
	*ACC*	*SN*	*SP*	AUC	*ACC*	*SN*	*SP*	AUC
**MNet-121**	88.9%	92.6%	87.2%	97.1%	84.6%	78.5%	88.0%	85.7%
**MNet-161**	91.3%	82.9%	95.3%	97.5%	82.1%	57.1%	96.0%	85.0%
**MNet-169**	85.0%	85.3%	84.9%	94.2%	82.1%	64.3%	92.0%	82.8%
**MNet-201**	87.4%	63.4%	98.8%	94.6%	76.9%	42.8%	96.0%	85.7%

*ACC*: overall accuracy; *SN*: sensitivity; *SP*: specificity; AUC: area under curve; MNet-121/161/169/201: M3D-DenseNet with 121/161/169/201 layers.

**Table 4:** Comparing the performance between single-modality and multi-modality model.

	Training Dataset				Validation Dataset			
	*ACC*	*SN*	*SP*	AUC	*ACC*	*SN*	*SP*	AUC
**SNet-T1**	67.7%	56.6%	92.4%	67.6%	64.1%	43.2%	80.2%	47.4%
**SNet-T2**	77.9%	82.9%	75.6%	87.5%	74.4%	78.6%	72.0%	81.6%
**SNet-T1Gd**	74.8%	65.9%	79.0%	81.0%	74.3%	50.0%	88.0%	74.6%
**SNet-FLAIR**	76.3%	31.7%	97.7%	81.0%	71.8%	35.7%	92.0%	72.6%
**MNet-121**	88.9%	92.6%	87.2%	97.1%	84.6%	78.5%	88.0%	85.7%

Single-modality 3D DenseNet with 121 layers; MNet: M3D-DenseNet with 121 layers.

**Table 5:** World Health Organization (WHO) grade status prediction performance.

	Training Dataset				Validation Dataset			
	*ACC*	*SN*	*SP*	AUC	*ACC*	*SN*	*SP*	AUC
**MNet-121**	75.2%	96.3%	42.3%	88.4%	80.0%	100%	46.1%	84.3%
**MNet-161**	75.9%	62.9%	96.2%	94.3%	77.1%	68.2%	92.3%	87.9%
**MNet-169**	91.7%	93.8%	88.5%	96.9%	85.7%	86.4%	84.6%	91.1%
**MNet-201**	90.2%	88.9%	92.3%	95.3%	91.4%	92.3%	92.3%	94.8%

M3D-DenseNet with 121/161/169/201 layers.
